# Evans Syndrome Presenting as an Atypical Complication of SARS-CoV-2 Vaccination

**DOI:** 10.7759/cureus.26602

**Published:** 2022-07-06

**Authors:** Marco De Felice, Giuliana Farina, Rosario Bianco, Giuseppe Monaco, Salvatore Iaccarino

**Affiliations:** 1 Hematology and Medical Oncology, AORN Sant'Anna and San Sebastiano, Caserta, ITA; 2 Clinical Oncology, Università degli Studi della Campania "Luigi Vanvitelli", Naples, ITA

**Keywords:** vaccination, sars-cov 2, immune thrombocytopenia, autoimmune haemolytic anaemia, evans syndrome

## Abstract

The coronavirus disease 2019 (COVID-19) pandemic, caused by severe acute respiratory syndrome coronavirus 2 (SARS-CoV-2), has drastically affected our daily lives, causing millions of deaths worldwide. The early and late complications of this infection are being increasingly revealed on a regular basis; however, an important brake on the spread and especially the lethality of the disease has been guaranteed by the introduction of mRNA-based and viral vector-based COVID-19 Vaccines. Also, an increasing number of adverse effects of the vaccination have been reported to specific pharmacovigilance boards, most of them totally non-serious events that are resolved within one to three days after the administration of the vaccine. In this report, we present a case of Evans syndrome (ES) secondary to SARS-CoV-2 vaccination in an 85-year-old male patient. To the best of our knowledge, this is the first case of ES caused by the COVID-19 vaccination to be reported in the literature.

## Introduction

Evans syndrome (ES) is defined as sequential or concomitant autoimmune hemolytic anemia (AIHA) and immune thrombocytopenia (ITP) due to warm antibodies, usually of IgG isotype and occasionally IgA, thus excluding cold agglutinins [1]. It is an extremely rare disease, with an annual incidence of 1.8/million person-years, and an annual prevalence of 21/million persons in the United States. ES is predominantly diagnosed during the fifth-sixth decades of life, with a female predominance and chronic course in more than 80% of patients, often with multiple relapses [2]. Its differential diagnosis is complex, involving thrombotic microangiopathies, anemia due to bleedings complicating ITP, cytopenia induced by viral infections such as cytomegalovirus (CMV), Epstein-Barr virus (EBV), human immunodeficiency virus (HIV), parvovirus B19, vitamin deficiencies, myelodysplastic syndromes, paroxysmal nocturnal hemoglobinuria, drug-induced cytopenia, and autoimmune disorders [3]. In 30-40% of cases, ES is secondary to another disease, most particularly hematologic malignancies and systemic lupus erythematosus. However, due to the ongoing coronavirus disease 2019 (COVID-19) pandemic, caused by severe acute respiratory syndrome coronavirus 2 (SARS-CoV-2), the condition has also been reported to be associated with COVID-19 infection, with different clinical patterns and management compared to primary ES [4-5]. In this report, we describe an atypical case of isolated secondary ES induced by the SARS-CoV-2 vaccine.

## Case presentation

An 85-year-old male presented to the emergency department (ED) of Sant'Anna and San Sebastiano Hospital in Caserta, Italy, in March 2021, due to the appearance of a large hematoma that extended from the left deltoid to the forearm, with the evidence of widespread ecchymosis on the right arm and legs. The scleras were icteric while conjunctivas were pale.

A complete physical examination revealed the following findings: temperature: 36.3 °C; heart rate: 85 beats/minute; blood pressure: 140/80 mmHg; respiratory rate: 15 breaths/minute; oxygen saturation: 96%. In anamnesis, the patient reported atrial fibrillation and hypertension, being treated with edoxaban 30 mg/day, furosemide 25 mg/day, bisoprolol 2.5 mg/day, and canrenone 50 mg/day, without any prior history of thrombocytopenia (Table 1).

**Table 1:** Biochemical parameters before receiving the SARS-CoV-2 vaccine SARS-CoV-2: severe acute respiratory syndrome coronavirus 2; LDH: lactate dehydrogenase

Biochemical parameter	Patient values	Reference range
Hemoglobin	14 g/dl	13-18
Mean corpuscular volume	88 fl	80-99
Mean corpuscular hemoglobin	31 pg/cell	26-32
Leukocytes	4,100 cells μL	4,000-11,000
Neutrophils	3,050 cells/μL	2,000-7,000
Lymphocytes	590 cells/μL	900-5,200
Platelets	180,000 cells/μL	150,000-400,000
LDH	220 UI/L	80-300
Total bilirubin	1 mg/dl	0.1-1.2
Direct bilirubin	0.2 mg/dl	<0.3
Haptoglobin	150 mg/dL	50-150
Direct Coombs test	0	0
Reticulocytes	2%	0.5-2.5

The patient had received the Comirnaty® SARS-CoV-2 vaccine at the beginning of March on his left arm, detecting first local ecchymosis 48 hours later. After seven days, he was admitted to our hospital. Blood counts in the ED showed severe thrombocytopenia at 8,000 cells/mmc, normochromic normocytic anemia with hemoglobin (Hb) of 10.0 g/dl, mean corpuscular volume (MCV) of 85 fl, mean corpuscular hemoglobin (MCH) of 29 pg/cell, leukocyte count of 4,950 cells/µL, neutrophils of 3,480 cells/µL, and lymphocytes of 790 cells/µL. Hemolysis tests were positive, with lactate dehydrogenase (LDH) of 400 UI/L, total bilirubin of 4 mg/dl, direct bilirubin of 2.8 mg/dl, reticulocyte count of 10%, haptoglobin of 20 g/L, and positive direct Coombs test (3+) (Table 2). Fibrinogen, D-dimer, prothrombin time (PT), activated partial thromboplastin time (aPTT), and C-reactive protein (CRP) were within normal ranges, and so were vitamin B12, folate, serum ferritin, and iron.

**Table 2:** Biochemical parameters in the emergency department LDH: lactate dehydrogenase

Biochemical parameter	Patient values	Reference range
Hemoglobin	10 g/dl	13-18
Mean corpuscular volume	85 fl	80-99
Mean corpuscular hemoglobin	29 pg/cell	26-32
Leukocytes	4,950 cells μL	4,000-11,000
Neutrophils	3,480 cells/μL	2,000-7,000
Lymphocytes	790 cells/μL	900-5,200
Platelets	8,000 cells/μL	150,000-400,000
LDH	400 UI/L	80-300
Total bilirubin	4 mg/dl	0.1-1.2
Direct bilirubin	2.8 mg/dl	<0.3
Haptoglobin	20 g/L	50-150
Direct Coombs test	3+	0
Reticulocytes	10%	0.5-2.5

Antibodies against coronavirus spike (S) protein were negative. Peripheral blood smear highlighted reticulocytes and nucleated RBCs, in the absence of schistocytes. There was no infiltrate on the chest X-ray, and the polymerase chain reaction (PCR) assay for COVID-19 was negative. Deep palpation of the abdomen revealed a spleen of parenchymatous consistency which, on ultrasound imaging, increased in volume, with a maximum diameter of 13 cm and homogeneous echogenicity.

**Figure 1:**
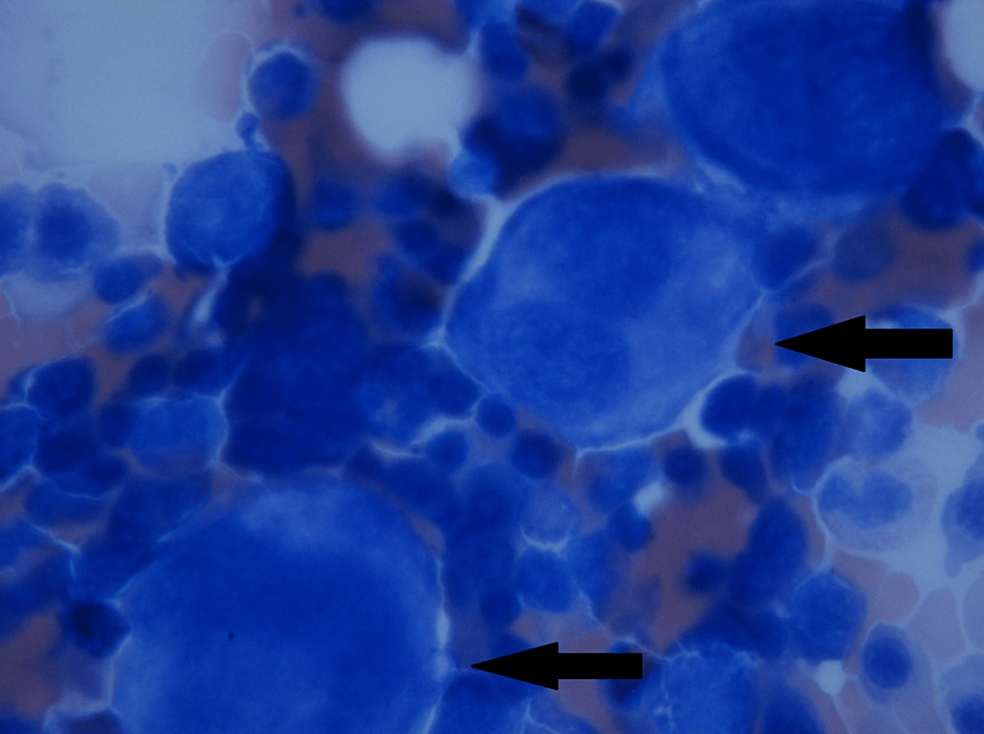
Bone marrow biopsy - image 1 Image showing diffuse abnormal and dysplastic megakaryocytes (black arrows)

**Figure 2:**
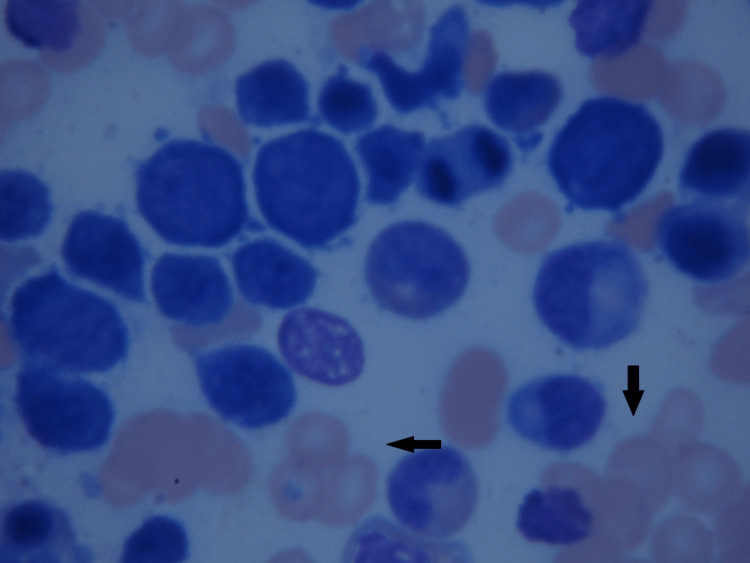
Bone marrow biopsy - image 2 Image showing abnormal hyperplasia erythroid (black arrows) and dysplastic megakaryocytes

A bone marrow aspiration was performed on the left iliac crest, which highlighted cellularity in the norm, erythroblasts series with elements in intermediate-final stages of maturation, and slight notes of dysplasia for nuclear-cytoplasmic mature asynchronism, mildly increased megakaryocyte series with some elements in terminal thrombocytopoiesis, and dysmorphic cells (Figures 1, 2). Based on these findings, a diagnosis of post-vaccination ES was made.

**Figure 3:**
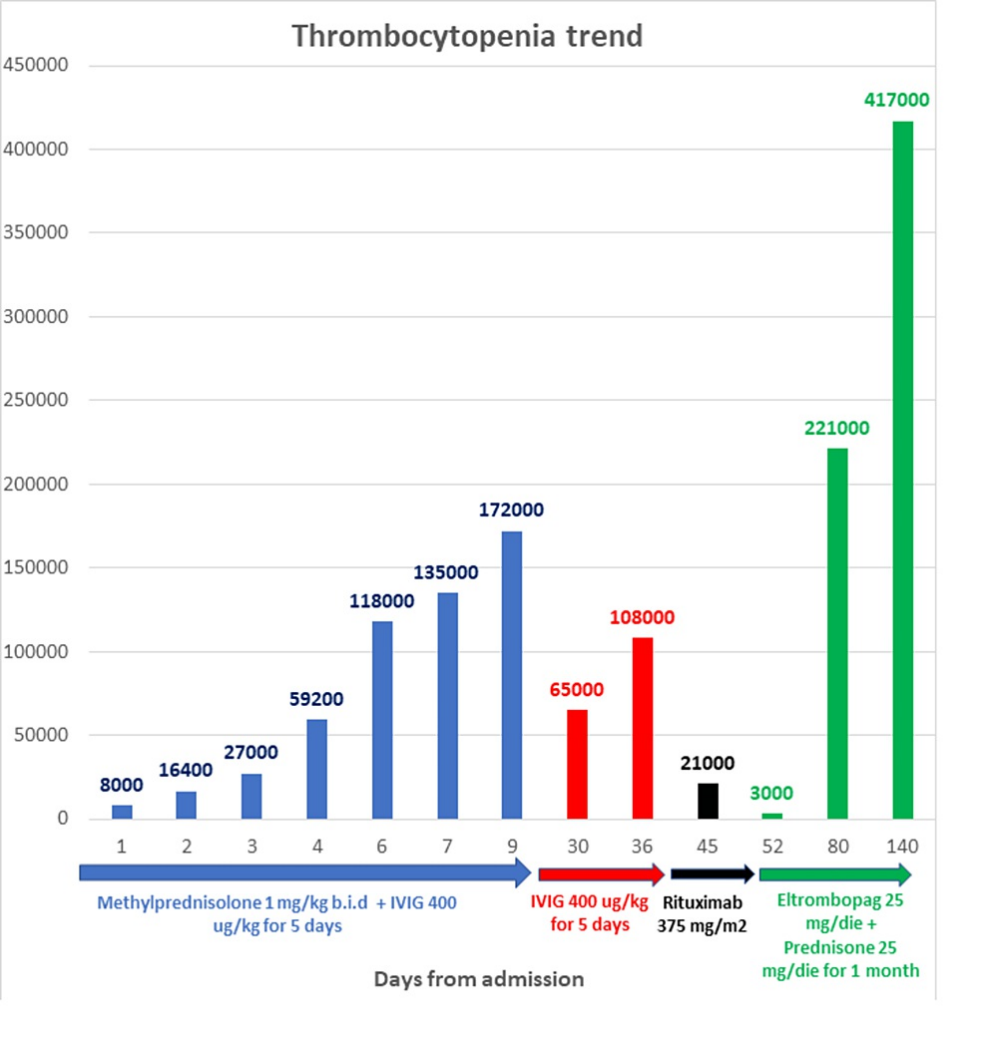
Thrombocytopenia trend and lines of treatment IVIG: intravenous immunoglobulin

We immediately began a combined intensive first-line treatment with methylprednisolone 1 mg/kg every 12 hours for four weeks and intravenous immunoglobulin (IVIG) 400 µg/kg for five days, associated with pantoprazole 40 mg/ev die. Gradually, the patient's platelet count normalized, as shown in Figure 3, along with his LDH and bilirubin levels. He was subsequently discharged on the 10th day of admission.

After three weeks, his Hb rose to 12.4 g/dl while platelets stabilized at 170,000 cells/µL; this prompted us to start steroid tapering as per clinical practice conventions. Unfortunately, his platelet level dropped to 65,000 cells/mmc and Hb to 10.5 g/dl, with a concomitant increase of total bilirubin to 2.2 mg/dl and LDH to 300 U/L. Hence, we decided to begin a new cycle of IVIG. SARS-CoV-2 antibodies continued to be negative.

After five IVIG administrations, the platelets rose to 108,000 cells/mmc and Hb to 11 g/dl; however, 10 days after stopping immunoglobulins, platelets dropped dramatically to 21,000 cells/mmc, and hence we rapidly undertook the third-line treatment of weekly rituximab 375 mg/m^2^.

Unfortunately, the patient had a further decline in platelet count to 3,000 cells/mmc, and we immediately decided to switch to eltrombopag 25 mg/day, thus excluding hemolysis and thrombosis, and prednisone 25 mg/day. Thirty days later, the platelets rose to 221,000 cells/mmc, with Hb stabilizing at 11.2 g/dl, and after three months, thrombocytes were at 417,000 cells/mmc. Prednisone was then tapered while eltrombopag therapy is still ongoing. Figure 3 depicts the patient's thrombocytopenia trend and lines of treatment.

## Discussion

ES is a rare disease that accounts for 7% of AIHA cases and around 2% of ITP. As for isolated autoimmune cytopenia (AIC), the determination of the primary or secondary nature of ES is important [3]. The association between ES and hematological malignancies, systemic lupus erythematosus, infections, or primary immune deficiencies could influence the management and modify the prognosis, which is poorer when compared to isolated AIC and the worst when associated with hematological malignancies [3]. Due to the rarity of the disease, the treatment is mostly extrapolated from what is recommended for isolated AIC. However, despite continuous progress in the management of AIC and a gradual increase in ES survival rates, the mortality rate still remains high, necessitating the need for an improvement in its management.

Corticosteroids represent the first line of therapy, usually at a daily dose of 1 mg/kg of prednisone, with a short course duration of three to four weeks for ES-thrombocytopenia and six months for ES-anaemia [6]. For life-threatening diseases, a bolus of methylprednisolone up to 15 mg/kg/day should be considered [6]. IVIG should be also considered along with corticosteroids for patients with severe thrombocytopenia (<30,000 cells/mmc) as emergency therapy, which would lead to a quicker increase in platelet counts. IVIG is much more effective in managing ITP than AIHA and it might increase the typical thrombosis risk of AIHA too [7]. Second-line treatments like rituximab, immunosuppressants, and splenectomy are recommended for both isolated AIHA and ITP, with an overall response rate ranging from 60% to 100% in various case series [3]. In specific situations such as autoimmune lymphoproliferative syndrome (ALPS), mycophenolate mofetil or sirolimus should be preferred [3]. Thrombopoietin receptor agonists (TPO-RA) have clearly demonstrated efficacy in isolated ITP with a response rate of 70-80%; however, a hypercoagulable state has been observed, especially in cases of concurrent ES with active hemolysis [8,9].

Among the hematological complications of SARS-CoV-2 infection, a systemic hyperinflammatory state resembling a secondary hemophagocytic lymphohistiocytosis and distinct coagulopathy taking after the hypercoagulable stage of disseminated intravascular coagulation (DIC) have been described [10]. SARS-CoV-2-mediated immune ITP could be attributed to an underlying immune dysregulation, susceptibility mutations in SOCS1, mechanisms of molecular mimicry, and epitope spreading [11]. The majority of ITP cases (71%) were found to be elderly individuals (aged >50 years), and 75% of cases had moderate-to-severe COVID-19. No bleeding manifestations were reported in 31% of cases at diagnosis and severe life-threatening bleeding was uncommon during the course of the disease. A good initial response to short-course glucocorticoids and IVIG has been described, while short-course TPO-RA has been used as a second-line agent in a few cases with no adverse events [12].

ES has been recently reported secondary to COVID-19 infection, with different clinical patterns and management compared to primary ES [10,11]. In fact, in cases of concomitant SARS-CoV-2 infection, ES treatment must be carefully balanced with the higher risk of thromboembolism associated with COVID-19, as reported by Li et al., and obviously the higher infectious risk linked to immunosuppressive treatment [5].

The clinical picture of moderate-to-severe thrombocytopenia and thrombotic complications at unusual sites, beginning approximately one to two weeks after vaccination against SARS-CoV-2, has been recently reported in connection with the ChAdOx1 nCov-19 vaccine, suggesting a disorder that clinically resembles severe heparin-induced thrombocytopenia, mediated by platelet-activating antibodies against PF4 [13]. Moreover, a case of extensive thrombosis associated with severe thrombocytopenia and DIC similar to autoimmune heparin-induced thrombocytopenia has been reported in a patient who had received the Ad26.COV2.S vaccine [14], suggesting that the rare occurrence of vaccine-induced immune thrombotic thrombocytopenia could be related to adenoviral vector vaccines, unlike messenger RNA-based vaccines.

Anyway, to our knowledge, this is not the first evidence of the association of ES with SARS-CoV-2 vaccination. Hidaka et al. have reported a new-onset ES with systemic lupus erythematosus after a second dose of BNT162b2 mRNA COVID-19 vaccination, quickly resolved with prednisolone 1 mg/kg/day [15]. Specifically, our case describes an isolated secondary ES after a single dose of SARS-CoV-2 vaccine, demonstrating a rapid resolution of AIHA although with the persistence of ITP, which generally tends to be more sensitive to immunosuppressive drugs than AIHA. After the failure of second-line treatment with rituximab, given the reduced risk of thrombosis in the absence of active AIHA, we proposed a third-line treatment with TPO-RA, which led to the rapid resolution of severe thrombocytopenia; however, the optimal duration of the therapy is not known, and hence the patient is still continuing therapy with eltrombopag.

## Conclusions

Our experience with this case highlights how maximum attention must be paid to the complications related to innovative mRNA-based vaccination too. Patients who do not develop antibodies against S protein may be at higher risk of developing autoimmune diseases due to a putative predisposition for a heterologous immunological response. Considering the severe prognosis and often the difficulty in managing these complications, prompt and early identification of risk factors is essential to successfully manage this pandemic-related emergency. Pharmacovigilance plays an essential role given that mass vaccination must be carried out and related adverse events will continue to be reported.
